# Biological Agents’ Adverse Events in Jordanian Childhood Rheumatic Diseases: A Single-Center Experience

**DOI:** 10.7759/cureus.78122

**Published:** 2025-01-28

**Authors:** Motasem O Alsuweiti, Heba Q Alma’aitah, Hamza M Alnsour, Bushra A Alwraikat, Ali I Alzyoud, Mohammed Nobani, Raed M Alzyoud

**Affiliations:** 1 Immunology, Allergy and Rheumatology, Royal Medical Services, Queen Rania Children's Hospital, Amman, JOR; 2 Ophthalmology, Royal Medical Services, Princess Haya Military Hospital, Ajloun, JOR; 3 Pediatric Dentistry, Royal Medical Services, Prince Hashem bin Abdullah Military Hospital, Aqaba, JOR; 4 Immunology, Allergy and Rheumatology, Royal Medical Services, Queen Rania Children’s Hospital, Amman, JOR

**Keywords:** adverse events, biological therapy, pediatric rheumatology, queen rania children's hospital, rheumatic diseases

## Abstract

Rheumatic diseases encompass a heterogeneous group of autoimmune and autoinflammatory disorders affecting joints, muscles, and connective tissue, with juvenile idiopathic arthritis (JIA) being the most prevalent among children. The introduction of biological agents in the treatment of childhood rheumatic diseases has significantly improved outcomes and quality of life. However, there is limited data on the use of biological agents in Jordanian children with these conditions. This study aims to evaluate the clinical indications for initiating biological agents and investigate the safety profile of biological therapy in Jordanian children. This retrospective study was conducted at Queen Rania Children's Hospital, including children (under 14 years of age) diagnosed with rheumatic diseases between January 2019 and December 2023. Data collected and reviewed included demographic characteristics, diagnosis, concomitant use of disease-modifying conventional synthetic antirheumatic drugs (csDMARD), indications for starting biological agents, age at initiation of therapy, and reported side effects during treatment. A total of 177 patients (57.6% females) with various rheumatic disorders were treated with biological therapy. The mean age at diagnosis was 7 years (range: 1-7 years), while the mean age at therapy initiation was 8.6 years (range: 4.2-14 years). JIA was the most common diagnosis in 110 patients (62%), followed by non-infectious uveitis in 18 patients (10.1%) and autoinflammatory disorders in 17 patients (9.6%). Concomitant therapy with DMARDs and corticosteroids was employed in 92.6% of cases (164 patients).

A total of 290 biological treatments were administered, including infliximab (93; 32%), etanercept (71; 24.4%), adalimumab (48; 16.5%), tocilizumab (41; 14.1%), and anakinra (13; 4.4%). In 66 patients (37.2%), the biological agent was switched, primarily due to inefficacy (56 patients, 84.7%) or adverse events (10 patients, 15.3%). A total of 64 adverse events (36.1%) were reported, the most common being infections (29; 45.3%), local reactions (11; 17.1%), and infusion-related reactions (10; 15.6%). This retrospective study highlights the importance of evaluating the efficacy and safety of biological agents in treating childhood rheumatic diseases. The findings can help optimize treatment strategies and improve patient outcomes.

## Introduction

Childhood rheumatic diseases encompass diverse conditions with distinct clinical presentations, including juvenile idiopathic arthritis (JIA), juvenile dermatomyositis (JDM), systemic lupus erythematosus (SLE), and systemic vasculitis. Conventional disease-modifying synthetic antirheumatic drugs (csDMARDs) such as methotrexate and leflunomide are often used as first-line treatments [1,2]. While many patients respond to these therapies, some experience suboptimal efficacy over time, poor tolerability, or significant side effects, necessitating the use of biological agents.

Biological agents have emerged as effective and relatively safe therapies for managing various rheumatic conditions [3]. However, data on the long-term safety of biological agents in children from the Middle East remain limited [4]. This study aims to address this gap by evaluating the clinical indications for initiating biological agents and assessing their safety profile in children treated at the pediatric rheumatology clinic of Queen Rania Children’s Hospital.

## Materials and methods

This is a retrospective study investigating adverse events of biological agents in children younger than 14 years of age, who were diagnosed with autoinflammatory disorders, JIA, JDM, SLE, non-infectious uveitis, and systemic vasculitis (Kawasaki disease, IgA vasculitis, Wegener’s granulomatosis, leukocytoclastic vasculitis, and Behçet’s disease), between January 2019 and December 2023. Patients who were diagnosed and followed in the pediatric rheumatology department in Queen Rania Children’s Hospital were included in the study, while patients who developed mild adverse reactions and were treated in local health care centers were excluded. The diagnosis of patients and treatment options were determined by pediatric rheumatologists accordingly.

The data of all patients was retrieved from their medical records, encompassing demographic information such as age and gender, age at the time of diagnosis, concurrent medications, duration of biological treatment, and documented adverse events. Before commencing the study, all of the patients’ parents provided written informed consent. Ethical approval was obtained from the local ethical committee of the Royal Medical Services.

The participants received a variety of biological agents including TNF-α inhibitors such as etanercept, adalimumab, golimumab, and infliximab. Additionally, they were also given an IL-1 receptor antagonist (anakinra), a humanized monoclonal antibody that targets the IL-6 receptor (tocilizumab), and a humanized monoclonal antibody that targets CD20 (rituximab).

Pediatric rheumatologists carefully chose the appropriate biological agents based on the specific type and severity of the disease, and made the decision to alter or cease treatment based on adverse reactions or the patient achieving remission. The patients receiving biological agents were followed up regularly monthly by pediatric rheumatologists for at least six months.

The medical records of the patients were reviewed for adverse events and serious adverse events, which contained detailed information gathered through symptom inquiries, physical examinations, and regular laboratory testing. These assessments were conducted every three months during routine follow-up appointments. Serious adverse events were categorized as instances that led to death, posed a threat to life, necessitated hospitalization, or caused enduring and substantial impairment or disability.

Upper respiratory tract infections (URTIs) such as rhinosinusitis, pharyngitis, laryngitis, laryngotracheitis, and otitis media were managed at outpatient facilities. Local injection site reactions were characterized by the presence of erythema, pruritus, discomfort, edema, and a sensation of heat at the injection site.

Descriptive statistics were used to summarize the data, with continuous variables presented as mean ± standard deviation (SD) and categorical variables as frequencies and percentages.

## Results

A total of 177 patients (57.6% female individuals) with various rheumatic disorders were treated with 290 biological agents. Among these, 111 patients (62.7%) were treated with a single biological agent, 43 patients (24.3%) with two agents, and 23 patients (13%) with more than two agents. Patient demographics and the distribution of biological agents are detailed in Table 1.

**Table 1:** Patient demographics and number of biological agents.

Number of patients (M: F)	177 (75: 102)
Mean age at diagnosis (range) years	7 (1 - 7)
Mean age at starting biological agent (range) years	8.6 (4.2 - 14)
Mean treatment duration (range) months	16.3 (3 - 60)
Total biological agents	290
Patients used one biological agent	111 (62.7%)
Patients used two biological agents	43 (24.3%)
Patients used three biological agents	9 (5%)
Patients used four biological agents	9 (5%)
Patients used five biological agents	5 (3%)

All patients with JIA were treated with methotrexate, and 13 of these patients also received leflunomide. Radiological and laboratory assessments, including chest X-rays, hepatitis profiles, and tuberculin skin tests were conducted for all patients before initiating biological treatment.

The 290 biological treatments comprised 93 (32%) infliximab, 71 (24.4%) etanercept, 48 (16.5%) adalimumab, 41 (14.1%) tocilizumab, 13 (4.4%) anakinra, 10 (3.4%) secukinumab, 8 (2.7%) golimumab, and three treatments each (1%) of tofacitinib and rituximab. Frequencies of the biological agents and the spectrum of diagnosis are demonstrated in Table 2.

**Table 2:** Frequencies of biological agents and spectrum of diagnosis. JIA: juvenile idiopathic arthritis, IBD: inflammatory bowel disease, FMF: familial Mediterranean fever, MVK: mevalonate kinase, JDM: juvenile dermatomyositis.

Diagnosis	Frequency	Etanercept N = (71)	Infliximab N = (93)	Adalimumab N = (48)	Golimumab N = (8)	Tocilizumab N = (41)	Secukinumab N = (10)	Tofacitinib N = (3)	Anakinra N = (13)	Rituximab N = (3)
JIA	110	56	48	24	8	26	8	2	4	1
Oligoarticular	46	26	24	14	0	4	0	1	0	0
Polyarticular	15	9	6	1	3	0	0	0	0	0
Systemic onset	31	10	11	6	4	21	1	1	3	1
Psoriatic	6	6	3	0	0	1	1	0	1	0
Enthesitis related	9	3	3	1	1	0	5	0	0	0
IBD related	3	2	1	2	0	0	1	0	0	0
Autoinflammatory	17	8	14	5	0	7	0	0	6	0
FMF	4	3	1	0	0	0	0	0	0	0
MVK	6	3	5	3	0	5	0	0	4	0
Others	7	2	8	2	0	2	0	0	2	0
Vasculitis	13	0	7	7	0	0	0	0	0	0
Uveitis	18	0	13	9	0	0	0	0	0	0
JDM	2	0	2	0	0	1	0	1	0	1
Others	17	7	9	3	0	7	2	0	3	1

Most patients (111; 62.7%) initiated biological treatment due to disease activity despite treatment with cDMARD, while 66 patients (37.3%) began treatment after the failure of a previous biological agent. Sixty-six patients (37.2%) switched biological agents; due to inefficacy (56 patients; 85%) or adverse events (10 patients; 15%). Among these, 43 patients (65%) were treated with two sequential biological agents, and 23 patients (35%) used up to five agents.

A total of 28 patients (16%) reported adverse events, resulting in a total of 64 adverse events associated with biological treatment. Table 3 outlines the types and frequencies of these events.

**Table 3:** Adverse events reported in children treated with biological agents.

	Etanercept	Infliximab	Adalimumab	Golimumab	Tocilizumab	Secukinumab	Anakinra	Tofacitinib	Rituximab	Total
No. of patients	71	93	48	8	41	10	13	3	3	290
Total adverse events	13	16	8	1	14	3	6	0	3	64 (100%)
Infusion-related	0	5	0	0	4	0	0	0	1	10 (15.6%)
Local reaction	5	0	1	1	0	1	3	0	0	11 (17.1%)
Infection	6	7	4	0	6	1	3	0	2	29 (45.3%)
Leukopenia	0	0	1	0	4	0	0	0	0	5 (7.8%)
Transaminitis	0	3	1	0	0	0	0	0	0	4 (6.2%)
Others	2	1	1	0	0	1	0	0	0	5 (7.8%)

Minor adverse events accounted for 54 cases (84%), while 10 cases (16%) were classified as major. The most common adverse events were infections, occurring in 29 patients (45.3%), primarily upper respiratory tract infections (URTIs) that were managed with symptomatic treatment and oral antibiotics.

Local reactions to subcutaneous injections, characterized by redness and pain, were observed in 11 patients (17.1%). One severe case treated with anakinra led to soft tissue cellulitis, requiring hospitalization and intravenous antibiotics. Anaphylaxis-like reactions occurred in 10 patients (15.6%)-five from infliximab, four from tocilizumab, and one from rituximab-necessitating treatment discontinuation. No mortality or malignancy related to biological agents was reported.

## Discussion

The advent of biological therapies targeting specific pro-inflammatory cytokines (tumor necrosis factor (TNF), interleukin (IL)-6, and IL-1), which are responsible for JIA’s pathogenesis, revolutionized the use of biological agents in the treatment of the disease [5]. Consequently, the use of biological agents is emerging as a key treatment alternative for the traditional management of JIA. However, existing information regarding the use of biological medications in pediatric patients with rheumatic conditions, aside from JIA, is primarily based on observational studies and case reports, Breda L et al. [6]. In the present study, JIA was identified as the most common diagnosis, with anti-TNF alpha being the most frequently prescribed biological agent for treatment. Moreover, the frequent use of unapproved biological agents for various diseases such as JDM and autoinflammatory disorders in this cohort was widespread, including the use of rituximab and infliximab for JIA.

Children with rheumatic diseases often require long-term treatment. In cases where anti-TNF therapy fails or causes toxicity, switching to a different biological agent is a common strategy. However, the risks associated with switching remain unclear [7,8]. In this study, 66 patients (37.3%) switched biological agents, with 43 patients (24.3%) using two agents sequentially, and 23 patients (13%) using up to five agents. This subgroup represented the study group’s extreme part comprising individuals who did not respond to their previous biological treatment, with ten patients (15.1%) being unable to tolerate the initial therapy. While several published studies have shown favorable safety and tolerability profiles for different biological agents, satisfactory data on long-term safety in children with rheumatic diseases treated with biological agents, particularly from the Middle East, is scarce [3,4,6,9].

Although most adverse effects that have been reported in the literature are mild, such as URTIs and infusion-related reactions, serious effects including autoimmune events, malignancy, and opportunistic infections have also been documented in some studies [4,10]. In the present study, 64 adverse events were associated with biological treatment, with the majority being infections (45.3%); classified as minor events. The occurrence of infections in the patients was expected, and these infections being mild, did not necessitate treatment modification or hospitalization, consistent with findings from a published study by Balcı S et al. [11]. Some studies suggest that the infection risk associated with TNF inhibitors is comparable to that of non-biological DMARDs, while others highlight an increased risk of serious infections with TNF inhibitor treatment [12,13].

Injection site reactions were observed in 11 patients (17.1%), with 10 reporting mild redness that did not require switching to another biological agent. One patient developed severe local soft tissue cellulitis due to anakinra, necessitating hospitalization and antibiotic treatment. In this study, injection site reactions were higher than those reported by Swart et al. [14], which may be attributed to the higher number of injectable biological agents compared to intravenous agents in the current study.

Another adverse event reported in our study was an infusion-related anaphylaxis-like reaction, occurring in 10 patients (15.6%), necessitating treatment termination. This finding was higher than reported by Koç et al., who documented a 5.5% incidence of infusion-related reactions, potentially due to the higher number of patients (47.2%) receiving intravenous biological agents [15]. Additionally, there is significant concern regarding the potentially higher risk of malignancy associated with the use of TNF inhibitors in children compared to the general population [16]. Children with rheumatic diseases have a higher likelihood of developing malignancy than the general population, but the use of TNF inhibitors does not appear to significantly increase this risk [17]. Notably, no patients in our study developed malignancy throughout the entire follow-up period.

Our study has several limitations, with one of them being a retrospective study. Additionally, it was performed in a single center which did not enable coverage of the relevant population so commentators could comment more on the adverse effects of the biological agent in context. Finally, the fact that all these patients were seen and followed up at one tertiary referral center may have biased the clinical classification because some of the patients with mild adverse events who did not attend were excluded.

## Conclusions

In conclusion, to the best of our knowledge, this is the first study from Jordan addressing the long-term safety of biological agents in pediatric patients with rheumatic diseases. This study’s findings highlight the diversity and favorable safety profile of these agents in children. As per the present study, the side effect that was the most in patients taking biological therapy is infection (upper respiratory tract infections). Serious adverse events, accrued in a total of 15.6%. Even though serious adverse events are infrequent in the clinical setting and can usually be easily managed with either cessation of the therapy or with the treatment of the adverse events, we recommend that patients who are being treated with biological agents be carefully monitored for the occurrence of these potential adverse events.
